# Valorization of *Pennisetum setaceum*: From Invasive Plant to Fiber Reinforcement of Injected Composites

**DOI:** 10.3390/plants12091777

**Published:** 2023-04-26

**Authors:** Patricia Cabrera-García, María Dolores Marrero, Antonio Nizardo Benítez, Rubén Paz

**Affiliations:** 1 Departamento de Ingeniería de Procesos, Universidad de Las Palmas de Gran Canaria, Las Palmas de Gran Canaria, 35017 Las Palmas, Spain; 2 Departamento de Ingeniería Mecánica, Universidad de Las Palmas de Gran Canaria, Las Palmas de Gran Canaria, 35017 Las Palmas, Spain

**Keywords:** *Pennisetum setaceum*, invasive species, waste valorization, natural fibers, chemical treatment, injected composite

## Abstract

During the control campaigns of *Pennisetum setaceum* (invasive species widespread worldwide), the generated waste has accumulated in landfills. This study investigates its use to obtain *P. setaceum* fibers for their application as reinforcement of polymeric materials for injection molding, thus facilitating and promoting alternatives for the long-term sustainable management of *P. setaceum*. The extracted fibers were treated with alkaline, silane, acetic acid, and combined alkaline and silane treatments. Different composites with 20 and 40 wt% of fiber were extruded, and test samples were obtained by injection molding using recycled polyethylene as matrix. The composition of the fibers was determined by gravimetric methods, and contrasted with the analysis of the functional chemical groups using Fourier Transform Infrared Spectroscopy. Increases of up to 47% in the cellulose content of the treated fiber were observed. The thermal degradation was also evaluated using thermogravimetric analysis, which determined an increase in the degradation temperature, from 194 to 230 °C, after the combined alkaline–silane treatment. In order to analyze the differences in the composites, tensile, flexural, and impact properties were evaluated; in addition, differential scanning calorimetry was performed. Regarding the flexural behavior, it was possible to improve the flexural modulus up to 276% compared with that of the unreinforced polymer.

## 1. Introduction

Invasive species are characterized by their easy reproduction, as well as their high resistance to adverse conditions. For these reasons, they compete with endemic species of new areas to obtain the necessary sources of water and nutrients [1]. In addition to their influence on biodiversity, invasive species also have economic and social repercussions [2]. Due to the danger that the presence of these species entails, preventive actions are required in order to avoid reaching an uncontrolled spread. Among the measures proposed by some official sources, it is worth highlighting EU regulation 1143/2014, which includes restrictions on the maintenance, import, sale, reproduction, and cultivation of a list of Invasive Alien Species of Union concern (the Union list). One of the flora species included in this list is *Pennisetum setaceum* (Forssk) Chiov., which has recently been renamed as *Cenchrus setaceus* (Forssk.) Morrone [3].

*P. setaceum*, commonly known as fountain grass, is a perennial bunch grass native to Mediterranean parts of North Africa and the Middle East [4]. It can reach 130 cm in height, with inflorescences of 6–30 cm long. It is fire resistant and exhibits a high phenotypic plasticity, so it is capable of adapting to extreme drought or extra water and nutrient resources [1]. The fountain grass plant expands by dispersing seeds with the wind, as well as with vehicles [5], so its propagation along roads is very characteristic [6]. The presence of this plant has been reported in countries on all continents [7].

To deal with the presence of this widespread plant, there are two fundamental approaches: the use of the plants without their elimination, or after their elimination. Among the first use, the cultivation of these plants in wetlands built for the treatment of wastewater has been investigated [8], as well as electrogenesis mediated by the rhizosphere of the plants [9]. Regarding the second use, it stands out for potential application as a natural aggregate for cement mortars (up to 30 wt% of fiber), using treatment with hot water [10]. However, this previous work concluded that a chemical treatment was needed to improve the compatibility between the cement and the fiber. It is also worth noting its potential application in the manufacture of adobes for modern constructions (up to 8 wt% of fiber) [11]. Finally, research has also been carried out to obtain composites by rotational molding with fibers from this invasive species, which has been shown to add up to 20 wt% of fiber [12]. In this case, the composites with untreated fibers were compared to those with fibers treated with 1 M alkaline treatment, and evaluating the mechanical properties of the composites obtained.

In general, research on fibers is quite widespread, since it is very useful in multiple sectors, including the textile industry [13,14], construction [15], medicine [16], and the aerospace industry [17], among others. Specifically, the manufacture of plastic composites using natural plant fibers has become relevant because of the advantages of those composites compared with man-made fiber composites [18]. The most used man-made fibers for composite manufacturing are glass fiber, aramid fiber (Kevlar), and carbon fiber [19]. Compared with these synthetic fibers, the use of natural plant fibers is more economical, since their production requires a low energy cost [20]. However, the enhancement and industrialization of the extraction processes of some natural fibers is still a challenge that needs to be addressed in order to take advantage of the reduced energy required. In addition, they are less abrasive with the equipment and more eco-friendly because they are totally biodegradable [21].

The most common plant fibers used as reinforcement of polymeric composite materials are from kenaf, flax, hemp, jute, and sisal, because of their availability [22]; however, in recent studies, the use of less common plant fibers is being investigated [23]. The plant fiber extraction process depends on the type of plant. In a simplified description, three forms of fiber extraction are used: mechanical methods (manually, using rollers, mill, or hammer), chemical methods (immersing the fibers in acid, alkaline, or enzymatic solutions), and retting (using biological fermentation treatments) [24,25]. Its final diameter is highly dependent on the extraction method used. When the selected method is retting, the average diameter obtained is usually around 58 µm, and if ball milling is used, it is around 9 µm. The level of drying of the plant also influences the outcome, since fiber diameters of 18.6 µm and 63 µm have been reported when disc milling was used with green leaves and dry leaves, respectively [26]. Regarding composition, plant fibers are mainly constituted by cellulose, hemicellulose, and lignin, and, to a lesser extent, extractables, pectin, wax, and ash. Cellulose, which is usually the main compound, is crystalline, and the rest are amorphous components that negatively affect the adhesion between the fibers and matrix [27,28,29]. Thus, the fibers with low cellulose content produce worse quality composites. Furthermore, plant fibers have low thermal resistance, since hemicellulose begins to degrade at 180 °C, contrasted with cellulose and lignin at 240 °C and 280 °C, respectively [30]. In order to solve this challenge, the application of treatments on the fibers allows obtaining improvements both in thermal degradation and in matrix fiber compatibility, achieving a composite with better final properties [31]. Physical treatments, using sophisticated equipment, contribute to a cleaner surface, enhancing the surface characteristics and thus improving the fiber–matrix bonding [32]. The most common physical treatments are corona treatment [33], plasma treatment [34], and UV treatment [35], among others. Regarding chemical treatments, they reduce the OH- functional groups of fiber surfaces, modify the microstructure, and improve the surface roughness [36] obtaining better interaction between fiber and matrix. The most used chemical treatments are alkaline treatment [37,38], silane treatment [39], acetylation treatment [40], peroxide treatment [41], and bleaching treatment [42], among others.

The application of alkaline treatment 1 M on *P. setaceum* fiber has already been evaluated [12], achieving an improvement on the flexural and tensile properties of high-density polyethylene (HDPE) composites manufactured by rotational molding. Other investigations about alkaline treatment have been carried out with species of the same family. Ridzuan et al. [43] studied how alkaline treatment at different concentrations affects the fibers from *Pennisetum purpureum*. After testing with concentrations between 5 and 15%, it was found that in all cases, the initial temperature of fiber degradation increased by 13.7%. However, regarding the mechanical properties, the highest tensile strength of the fiber was obtained in the case of the 5% treatment. That is because, with higher concentrations, an excess of delignification occurred. On the other hand, publications about hemp fibers treated with 1 and 5% alkaline treatment reported losses in tensile strength of 14% and 24%, respectively [44]. Based on the results of the aforementioned research, treatment optimization for each species is required due to the great variability from one species to another. Regarding silane treatment, the applications of different silane molecules with concentrations between 0.1 and 5% have been evaluated for the treatment of kenaf fiber to be used as reinforcement of polypropylene (PP) or unsaturated polyester (UPE) composites [45]. Silane treatment with γ-methacryloxypropyltrimethoxysilane molecules enhanced interfacial shear strength up to 72%. A combined alkaline and silane treatment was also evaluated. Jute fibers treated with 2% alkaline treatment and silane with concentrations between 0.1 and 0.5% were used to obtain composites with polyester resin [46]. The mentioned study improved the mechanical properties of the composite when compared to the case of fiber treated with only the alkaline treatment. Regarding the acetic acid treatment, it was evaluated by subjecting *P. purpureum* fiber to acetic acid solutions with concentrations between 5 and 15%. As a result, increments of 16% in cellulose content, as well as improvement in thermal properties, were reported [47].

In the specific case of Spain, *P. setaceum* was introduced in 1940, becoming an invasive plant, especially in the Canary Islands, where it is known as “rabogato” [6]. In this context, control and eradication campaigns are currently applied to avoid the spreading of *P. setaceum*. However, these campaigns generate a large amount of waste, which accumulates in landfills. As an alternative, the present work proposes the valorization of this waste as a raw material for the manufacturing of more environmentally friendly composites by injection molding, using the obtained *P. setaceum* fiber as reinforcement and recycled high-density polyethylene as matrix. This manufacturing method is the most widely used method in commercial applications, since it allows for processing a large volume of parts with complex geometries in a very short time [48]. The use of injection molding in composites reinforced with *P. setaceum* fibers has not been evaluated so far. Another novelty consists in the chemical characterization of *P. setaceum* fibers before and after being treated with alkaline, acetic acid, and silane treatments, which have shown good results for enhancing the interfacial adhesion of matrix and other plant fibers. This alternative approach allows for more long-term sustainable control of invaded areas, since the funds invested in the eradication campaigns could be partially recovered, thanks to the valorization of the *P. setaceum* fiber into reinforced composite materials with multiple high-added-value applications.

## 2. Results and Discussion

### 2.1. Chemical Characterization

The results obtained after the chemical characterization of untreated and treated *P. setaceum* fibers are presented in Table 1. A, B and C correspond to three different individuals.

One of the most notable characteristics of natural plant fibers is the great variability of this material. Unlike synthetic fibers, natural plant fibers are exposed to different conditions during their life [24]. Among the factors that affect the fiber, the age of the plant, the growth environment, the harvest, the humidity, the quality of the soil, and the temperature stand out [21]. This difference is evident if the compositions of untreated fiber samples are compared (Table 1). As this variability between individuals makes it difficult to compare the different treatments in different individuals, the comparison was only carried out between untreated and treated fibers of each individual.

The composition of the acetic acid-treated fibers (PStb) was similar to that of untreated fibers (PS). As the fibers were characterized without eliminating extractables, the slight increase observed for all of the determined compounds was related to the reduction in the small proportion of initial extractables. In the literature, there are studies which coincide with this result [49], but there are also other authors who achieved a reduction in hemicellulose content after treatment [47]. Regarding the alkaline treatment (PSt and PSta), a reduction in amorphous substance (lignin and hemicellulose) content was observed, thus increasing the cellulose content, as reported by Guo et al. [50]. The hemicellulose reduction was greater when the concentration of alkaline treatment was higher, coinciding with the increase in cellulose, as stated in [51].

No significant variation in composition was observed in the fibers treated with silane (PStc). This also occurred if alkaline–silane (PStd) and alkaline fibers (PSt) were compared (both treatments led to similar results). Although some authors confirm that the silane molecule can degrade the most amorphous compounds [52,53,54], there are others who agree that the silane molecule only covers the fiber [55] to subsequently act as a bonding bridge between reinforcement and matrix.

### 2.2. Thermogravimetric Analysis (TGA)

**Figure 1:**
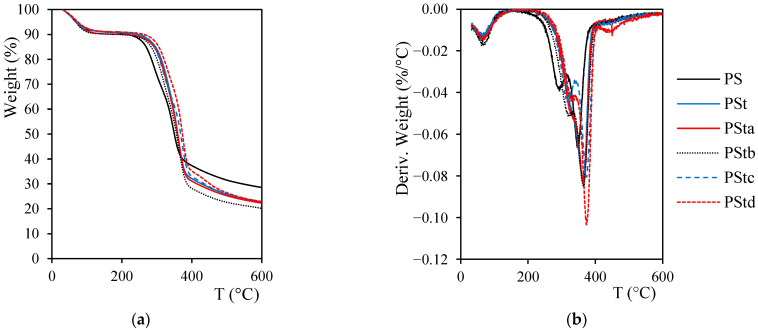
(**a**) Thermogravimetric analysis curves of untreated and treated *Pennisetum setaceum* fibers; (**b**) derivate of thermogravimetric curves of untreated and treated *Pennisetum setaceum* fibers. [PS: untreated fiber; PSt: 4% alkaline-treated fiber; PSta: 2% alkaline-treated fiber; PStb: acetic acid-treated fiber; PStc: silane-treated fiber; PStd: combined alkaline–silane-treated fiber].

The curves obtained after the TGA tests are presented in Figure 1. Initially, all of the fibers showed a slight weight reduction as the temperature increased up to 130 °C, with the highest loss peak at 70 °C. This first variation was mainly due to the presence of moisture in the fibers. The other two decomposition peaks correspond to hemicellulose (290–320 °C) and to lignin and cellulose (348–374 °C) [56]. In the derivate of thermogravimetric (DTG) curves, it can be observed that the peak corresponding to hemicellulose disappeared in the case of alkaline-treated 4% (PSt) and 2% (PSta) fibers. This matches with the results obtained in the chemical characterization of the fibers, as the hemicellulose content was reduced with the alkaline treatment.

For the determination of the extrusion and injection process parameters, it was also necessary to determine the temperature at which thermal degradation of the fiber begins. Table 2 shows the initial degradation temperature (T_i_), the temperature with the highest loss of weight (T_max_), and the final residue after TGA tests. The untreated fibers began to degrade at a temperature of 194 °C. After applying any of the treatments mentioned in this work, an improvement in the thermal resistance of the fibers was obtained, highlighting the combined alkaline–silane treatment. The temperature at which the fibers begin to decompose increased to 230 °C (18.5% higher). The improvements after the treatment with silane have also been reported by other authors [57,58], justified due to the adhesion of the silane molecules.

### 2.3. Fourier Transform Infrared Spectroscopy (FTIR)

**Figure 2:**
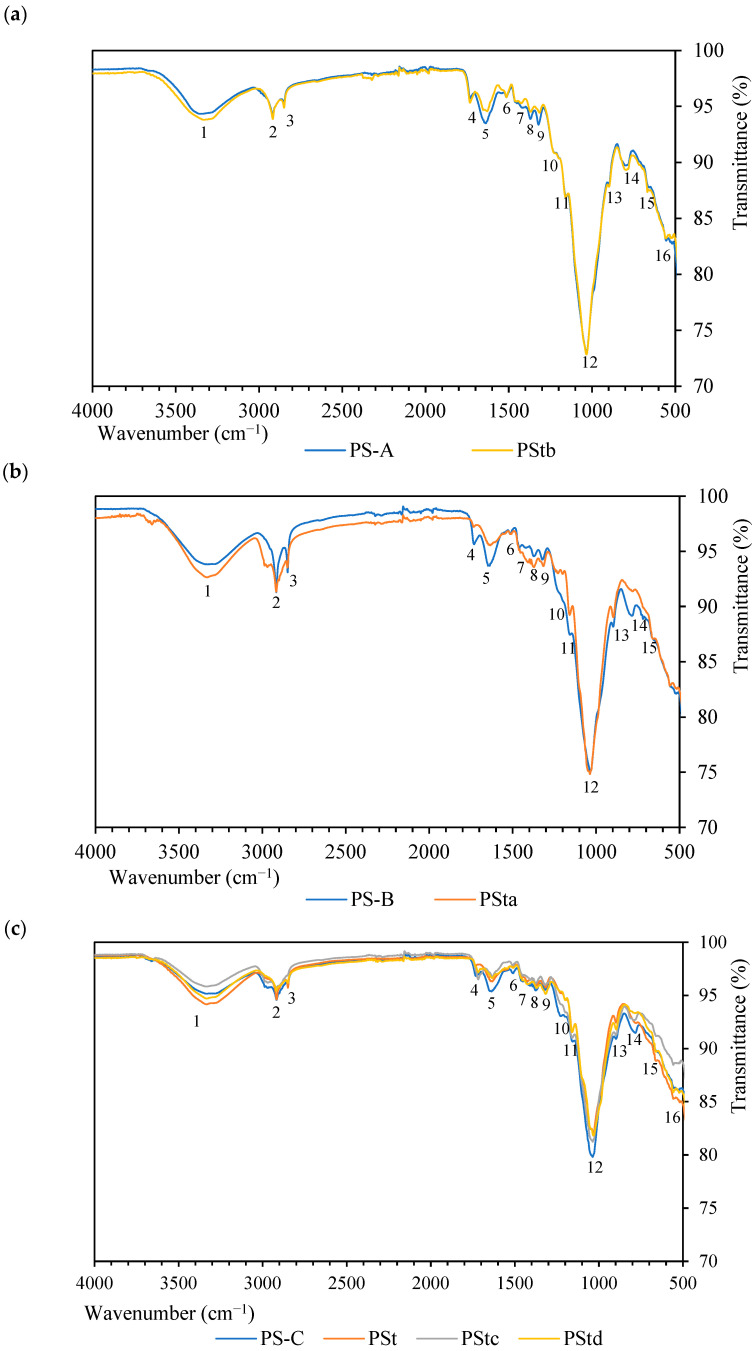
Fourier Transform Infrared Spectroscopy results of *Pennisetum setaceum* fibers: (**a**) individual A; (**b**) individual B; and (**c**) individual C.

Figure 2 depicts the results of the FTIR analyses of individuals A, B, and C, before and after the corresponding treatment.

Peak 1 obtained for a wavenumber of ~3330 cm^−1^ corresponds to -–OH stretching due to moisture [59]. Peaks 2 and 3 at ~2920 cm^−1^ and ~2850 cm^−1^ are related to the stretching and vibration of the C–H and CH_2_ groups, respectively, due to the presence of cellulose and organic compounds in general [60]. On the other hand, peaks 4 and 5 at wavenumbers ~1735 cm^−1^ and ~1650 cm^−1^, respectively, appear due to the C=O stretching of hemicellulose, in addition to the fatty acids present in oils [61]. Peaks 6 and 7 refer to the presence of lignin in the fiber, with 6 (~1520 cm^−1^) being related to the C=C stretching of the lignin benzene ring [43] and 7 (~1440 cm^−1^) to the C–O stretching of aromatic rings [60]. Peak 8 (~1380 cm^−1^) indicates the bending of the C–H functional group of cellulose and hemicellulose [59], while peak 9 (~1320 cm^−1^) corresponds to the phenol OH group of cellulose [62]. Regarding peak 10 (~1210 cm^−1^), it is related to hemicellulose, specifically with regard to the C–O bond [62]. Peaks 11 (~1160 cm^−1^) and 12 (~1040 cm^−1^) are related to lignin, specifically to the C–OC groups [27] and to the C–O hydroxyl and ether stretch [43], respectively. On the other hand, peak 13, located at the approximate wavenumber of ~900 cm^−1^, corresponds to the β-glycosidic bonds between the monosaccharides of cellulose [63]. Peak 14 (~800 cm^−1^) corresponds to the aromatic bending of lignin [49]. Finally, peak 15 (~670 cm^−1^) corresponds to the C–OH bending due to the presence of cellulose.

Figure 2a shows the spectra of individual A. The spectrum of untreated fibers was compared with the acetic acid-treated fiber one, only demonstrating reductions in peaks 5 and 7, that is, slight reductions in lignin and hemicellulose. On the other hand, Figure 2b shows the spectra of individual B, to compare how the 2% alkaline treatment affects the fiber. In this case, peaks 4, 5, and 10 almost completely disappear due to the removal of some of the hemicellulose during the treatment. A decrease in peak 7 was also observed, due to the partial removal of lignin. Figure 2c shows the spectra of individual C, comparing the untreated fibers with the fibers treated with 4% alkaline treatment, silane treatment, and combined alkaline and silane treatment. Regarding the alkaline treatment, reductions were observed in peaks 4, 5, and 10 due to the elimination of hemicellulose. On the other hand, in the case of treatment with silane, an increase in peak 4, related to the appearance of the MPS molecule used during the treatment, was detected, since this peak is related to the C=O functional group that appears in this compound. The same occurred in the case of combined alkaline and silane treatment, in addition to the reduction in peaks 5 and 10, as occurred in the alkaline treatment.

**Figure 3:**
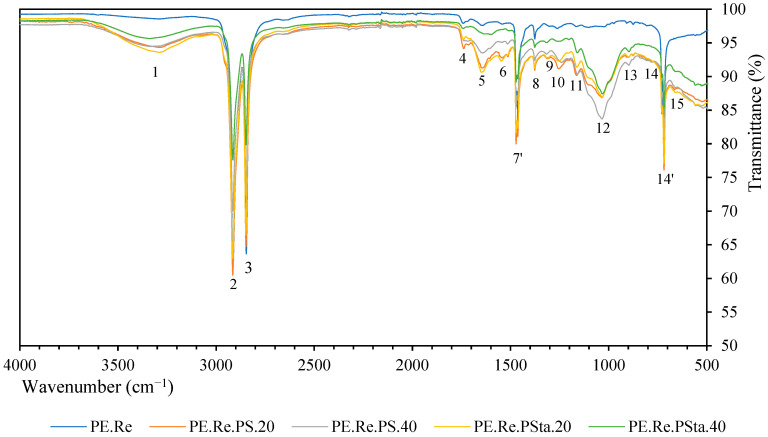
Fourier Transform Infrared Spectroscopy results of composite and recycled high-density polyethylene.

Regarding the FTIR analysis of the composite (Figure 3), the characteristic peaks of recycled high-density polyethylene are 2, 3, 7′, and 14′ (~2915 cm^−1^, ~2847 cm^−1^, ~1470 cm^−1^, and ~718 cm^−1^) [64]. The first two are related to the CH and CH_2_ bonds that also appear in the spectrum of the fiber in lesser intensity. On the other hand, peak 7′ corresponds to the C–C bond, while peak 14′ is related to the CH_2_ bond. As can be observed, when the fiber was added, the peaks described in Figure 2 also appeared. The intensity of the peaks, which were related to the fibers, was higher in the composites with 40 wt% of fibers.

### 2.4. Differential Scanning Calorimetry (DSC)

Table 3 shows the results after DSC tests of the composites. It includes crystallization temperature and enthalpy, as well as melting temperature and enthalpy determined during the second heating. In most of the cases, the addition of fiber (compared to neat polyethylene) slightly increased the crystallinity of the material. This indicates that the crystallization capacity improved by adding fiber, since the mobility of the polymer chains was restricted, allowing for the formation of smaller crystals [65]. In fact, with composite PE.Re.PSt.20, a crystallinity of 59.3% was achieved, compared to 54.7% crystallinity of PE.Re (an improvement of 8.4%).

### 2.5. Mechanical Tests

The tensile test results (Table 4) showed that the addition of untreated fiber to the polymer did not modify the tensile strength of the material. However, the fiber treatments improved the results of this parameter, achieving better results in the case of the alkaline treatment for the composites with 20 wt% of fiber (PE.Re.PSt.20 and PE.Re.PSta.20). Significant variations were also observed with respect to the neat polymer (PE.Re) in the case of silane and in the combined alkaline and silane treatment in the composites with 20 wt% of fiber (PE.Re.PStc.20 and PE.Re.PStd.20). Regarding the tensile modulus, the addition of fiber increased this parameter, to maximum in the cases of PE.Re.PStc.20 and PE.Re.Psta.40, with increases of 29.89% and 28.01%, respectively (compared with unreinforced recycled polyethylene PE.Re). According to the results of the statistical test, the only combinations with no significant variations compared to the neat polymer PE.Re were the untreated fiber composites (PE.Re.PS.20 and PE.Re.PS.40) and the composite with 40 wt% of fiber content and acetic acid treatment (PE.Re.PStb.40). Additionally, it was observed that the elongation at maximum tensile strength was lower for all of the composites compared to PE.Re. However, according to the statistical analysis, only some groups (PE.Re.PSt.20, PE.Re.PS.40, PE.Re.PSta.40, PE.Re.PStb.40, PE.Re.PStc.40, and PE.Re.PStd.40) showed significant differences compared to the neat polymer (PE.Re).

On the other hand, the addition of fiber made the material more resistant to bending (Table 5), achieving better results with treated fiber. Specifically, the PE.Re.PStd.40 composite had the best results, improving by 12.48% compared to the composite with the same percentage of untreated fiber (PE.Re.PS.40), and 69.16% compared to the neat matrix (PE.Re). According to the statistical test, all of the compounding materials showed significant differences compared to the neat polymer (PE.Re), with the exception of the composites with 20 wt% of untreated, alkaline-treated 2%, and acetic acid-treated fibers (PE.Re.PS.20, PE.Re.PSta.20, and PE.Re.PStb.20, respectively). The flexural modulus of the composites increased in all of the cases, especially in the composites with 40 wt% of fiber (PE.Re.PS.40, PE.Re.PSt.40, PE.Re.PSta.40, PE.Re.PStb.40, PE.Re.PStc.40, and PE.Re.PStd.40), and in the composite with 20 wt% of 4% alkaline treatment fiber (PE.Re.PSt.20). The highest improvement in flexural modulus was obtained in the case of PE.Re.PStd.40, with a 257.44% increase compared to the material without reinforcement (PE.Re). The improvements obtained in flexural parameters with silane-treated fibers were also reported by Sepe et al. [44]. It should also be noted that the elongation at maximum flexural strength was reduced in the composites compared to the neat recycled HDPE (PE.Re), especially in the composites with 40 wt% of fiber content (PE.Re.PS.40, PE.Re.PSt.40, PE.Re.PSta.40, PE.Re.PStb.40, PE.Re.PStc.40, and PE.Re.PStd.40). In general terms, the addition of fiber makes the material stiffer (higher tensile modulus, especially if the fiber was treated), but the increase in tensile strength is more limited. Therefore, composite materials achieve the maximum strength at lower elongations compared to the material without fiber.

Regarding the impact resistance results (Table 6), the addition of fiber reduced the resistance of the material. The reduction in impact strength agrees with the results reported of composites with *P. setaceum* by rotomolding [12]. It should be noted that the impact strength for the PE.Re.PS.40 composite was quite similar to that of PE.Re.PS.20. However, the composites with 40 wt% of treated fibers (PE.Re.PSt.40, PE.Re.PSta.40, PE.Re.PStb.40, PE.Re.PStc.40, and PE.Re.PStd.40) considerably reduced the impact resistance.

Supplementary Materials Figures S1 and S2 show the resistance (tensile, flexural, and impact strength) and the modulus of elasticity (tensile and flexural) of the composites, respectively, to facilitate the comparison under different types of loads. The addition of fiber to recycled HDPE reduces the impact resistance considerably. However, it significantly improves the resistance and the modulus of elasticity in bending.

## 3. Materials and Methods

### 3.1. Materials

*P. setaceum* plants were collected in Moya, the Canary Islands, Spain. Recycled high-density polyethylene (supplied by Plascan S.L., Las Palmas, Spain, in pellet format) was used as matrix. The chemical reagents used were sodium chlorite (80%, Honeywell Fluka^TM^, Charlotte, NC, USA), sodium hydroxide (≥98%, Honeywell Fluka^TM^, NC, USA), sulfuric acid (95–97%, Honeywell Fluka^TM^, NC, USA), 3-(Trimethoxysilyl)propyl methacrylate (98%, Sigma-Aldrich, St. Louis, MO, USA), and acetic acid glacial (99.8%, Labkem, Barcelona, Spain).

### 3.2. Fiber Extraction

*P. setaceum* plants were collected according to the local Technical Guidelines for the management, control, and elimination of “rabogato” (*Pennisetum setaceum*) (BOC Nº 120: Order of 13 June 2014). The first step was the elimination of the flowers of the plant, thus avoiding seed dispersion. These floral parts (and some seeds which had previously fallen to the ground) were carefully transferred to a bag. Subsequently, the leaves of the plants were collected by cutting them at the base, and then transported to be processed.

**Figure 4:**
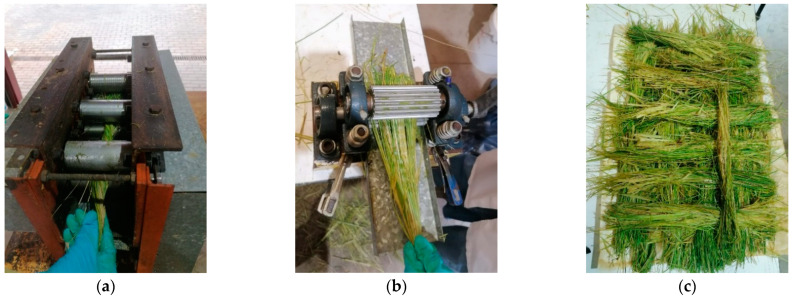
Extraction process of *Pennisetum setaceum* fibers: (**a**) Passing throw rollers; (**b**) Passing throw manual V-groove rollers; (**c**) Sun drying.

The fiber extraction was performed following an innovative procedure developed by our research group. This methodology was already applied in a previously published work by our team [12], but some additional details are described in the present paper in order to explain the intended purpose of the different stages of the process. Once the plants were harvested, the leaves were taken in bundles and processed in a lamination rolling machine with three types of paired rollers: knurled rollers (for good traction in the feeding pair of rollers), smooth rollers (for optimal compression of the leaves), and grooved rollers with sharp edges in the feeding direction (to separate the fibers between them) (Figure 4a). This combination of rollers caused a significant reduction in the water and filler compounds commonly found in the leaf. Therefore, after repeating this lamination process at least four times, the fibrous part of the leaves was obtained. Additionally, it was observed that the feeding of the bundles combined with manual twisting resulted in better fiber extraction results, as the twisting kept the fibers closer together in the rollers (avoiding spreading), thus increasing the thickness of the bundle and, consequently, producing a higher lamination force and improving the fiber extraction. Subsequently, the bundles of leaves were subjected to an additional lamination process with a manual pair of V-groove rollers (Figure 4b). These rollers were designed and manufactured with V-shaped grooves perpendicular to the feeding direction to mimic, in an automatic manner, the traditional process of stripping/breaking used for fibers such as hemp, flax, or jute. Therefore, this step improved the fiber separation. Finally, the fibers were stacked on a tray and sun dried (Figure 4c).

Long fiber tufts were cut using a cutting machine previously developed for the chopping of banana fiber [66]. Since *P. setaceum* fiber is less flexible than banana fiber, the conveyor belt (used for the feeding of material) and the first vertical rollers of the machine were disabled in order to reduce the risk of blockage. Thereafter, the *P. setaceum* was fed into the final rollers that drive the fiber to the cutting blade. With this process and adjusting the speed of the cutting blade and the feeding rollers, fibers with an average length between 1 and 2 mm were obtained. However, parts of the fibers were cut in excess, so they were sieved to removed particles of less than 75 µm.

### 3.3. Chemical Treatments

In order to improve the interfacial adhesion between the fiber and matrix, *P. setaceum* fibers were subjected to different treatments. To optimize the parameters of the treatments, some preliminary tests of composition determination (Fourier Transform Infrared Spectroscopy and gravimetric tests) were carried out based on the bibliography consulted. The final treatments used were acetylation treatment, i.e., soaking in 10% of acetic acid solution for 2 h [47]; alkaline treatment using 2% and 4% sodium hydroxide solution for 1 h [12]; silane treatment employing 3% of 3-(Trimethoxysilyl)propyl methacrylate (MPS), pre-hydrolyzed for 15 min in deionized water, for 1 h under agitation [45]; and a combination treatment of alkaline 4% and silane [46]. After all of the treatments, the fibers were washed with distilled water until the pH was neutralized, and dried in an oven at 60 ℃. One of the disadvantages of natural plant fibers is the great variability from one individual to another of the same species, even subjected to similar conditions. In order to evaluate how the treatment affects the fiber compounds, samples from 3 different individuals (A, B, and C) were processed. Individuals A, B, and C were processed to obtain fibers (some of the fibers of each individual were separated for the untreated composites). Afterwards, some of the fibers from individual A were treated with acetic acid treatment, while some of the fibers from individual B were treated with 2% alkaline treatment. Finally, some of the fibers from individual C were treated with 4% alkaline treatment, with silane treatment, and the combined alkaline–silane treatment. In this way, the real variation between the untreated and the treated fibers could be compared without being influenced by the difference that exists from one individual to another.

### 3.4. Composite Manufacture

The composites were prepared using the untreated or treated *P. setaceum* fibers mixed with the recycled HDPE. The fiber contents were 20 and 40 wt%. Compounding materials with all of the above-mentioned fiber treatments and contents were produced, except with 4% alkaline treatment in 40 wt% proportion, due to the poor processability of this compound in the extrusion process. The volume of the fiber bundle subjected to this treatment was greater due to fibrillation. Because of this volume increase, the fiber tended to accumulate at the tip of the extruder, hindering the extrusion and causing excessive pressure (above the limit of the machine).

Before mixing the materials for the compounding process, the fibers were previously dried at 105 °C for 12–24 h to remove moisture content. The HDPE matrix was also dried at 60 °C for the same time, following the provider’s instructions. The compounding extrusion was carried out with a co-rotating twin screw extruder (Thermo Scientific^TM^, Waltham, MA, USA, Process 11), including final pelletizing using a VariCut pelletizer.

**Figure 5:**
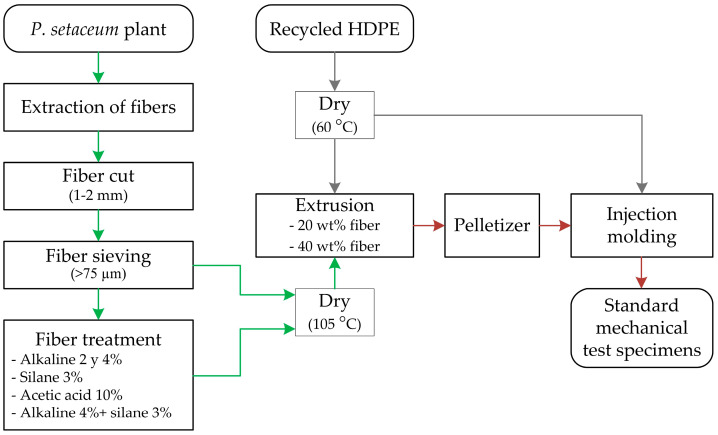
Process diagram for obtaining injected recycled high-density polyethylene (HDPE) composites reinforced with *Pennisetum setaceum* fiber.

Subsequently, the extruded pellets were injected (JSW J55AD-60H injection molding machine) in a mold cavity corresponding to standard mechanical test specimens (ISO 527). The injection parameters used were 175, 180, 185, and 190 °C in the screw heating zones, 1620 bar of injection pressure, 860 bar of holding pressure for 10 s, and 60 s of cooling time (no cooling or heating systems were used for the mold). Standard mechanical test specimens of unreinforced recycled HDPE (PE.Re) were also injected in order to evaluate the fiber influence on the material. Figure 5 shows a summary of the manufacturing process of the composites, from the extraction of the vegetable fiber to the obtaining of the standard mechanical test specimen.

The nomenclature used to identify the different composites was PE.Re.PS*Y*.*Z*, where *Y* refers to the treatment (abbreviations in Table 7) and *Z* is the fiber percentage in the composite.

### 3.5. Chemical Characterization

The chemical constituents of *P. setaceum* fibers were evaluated using gravimetric tests. Hydrolysis of constituents with 72% sulfuric acid solution was done to determine Klason lignin, according to ANSI/ASTM 1977a Standard test methods for lignin in wood [67]. The holocellulose content of each sample, which is the sum of cellulose and hemicellulose, was obtained using the Browning technique [68], degrading lignin content with sodium chlorite and acetic acid. Regarding cellulose content, it was determined from holocellulose, by degrading hemicellulose with sodium hydroxide, as stated in ANSI ASTM 1977b Standard test methods for alpha-cellulose in wood [69]. The hemicellulose content was calculated by determining the difference between holocellulose and cellulose contents. Finally, ash content was determined by introducing 1 g of sample into muffle at 550 °C for 24 h. Each sample was tested at least three times to confirm the result.

### 3.6. Thermogravimetric Analysis (TGA)

The thermal stability of untreated and treated *P. setaceum* fibers was evaluated using thermogravimetric analysis. Samples of 8–12 mg were characterized using a TGA 4000 (PerkinElmer, Waltham, MA, USA). The temperature was elevated from 30 to 600 °C at 10 °C/min rate. Samples from different individuals were evaluated by this method. Despite having obtained similar results, the average value of the three samples was calculated.

### 3.7. Fourier Transform Infrared Spectroscopy (FTIR)

Fibers and composite samples were also characterized using FTIR analysis. The equipment used to carry out this analysis was a Perkin Elmer Spectrum Two (PerkinElmer, Waltham, MA, USA). This apparatus is equipped with an attenuated total reflectance (ATR) device. The spectra were obtained as the average of 12 scans, from 4000 to 500 cm^−1^ at a resolution of 1 cm^−1^.

### 3.8. Differential Scanning Calorimetry (DSC)

Differential Scanning Calorimetry was carried out using a DSC 4000 (PerkinElmer, Waltham, MA, USA) under a nitrogen atmosphere. Samples of 10–12 mg of the manufactured composites were weighted in aluminum pans and were subjected to heating–cooling cycles at a 10 °C/min rate: heating from 30 to 200 °C, holding for 2 min at 200 °C, cooling from 200 to 30 °C, holding for 2 min at 30 °C, and heating from 30 to 200 °C. Four replicas of each sample were tested to obtain the melting and crystallization temperature and enthalpies. The equipment was configured to perform two heating cycles in order to eliminate the thermal history of the material from the first one, as is indicated in ISO 11357. The crystallinity value of the material was obtained using Equation (1).

(1) $$X_{c}(\%)=\frac{\Delta H_{m}\cdot 100}{(1-\%fiber)\cdot \Delta H_{m}^{0}}$$

where Δ*H_m_* is the melting temperature of the composite and Δ*H^0^_m_* is the melting temperature of the high-density polyethylene 100% crystalline, which is 293 J/g [70].

The results obtained from DSC were statistically analyzed using the Kruskal–Wallis test (‘kruskalwallis’ Matlab function) with 1% significance level. Therefore, when the *p*-value obtained was lower than 0.01, the alternative hypothesis was confirmed, which is that not all samples come from the same distribution. In those cases, a multi comparison test was also carried out (‘multcompare’ function in Matlab) to determine the groups that were statistically different from the control group, which was neat recycled HDPE (PE.Re). On the other hand, the box plots obtained from the Kruskal–Wallis test were used in order to eliminate the outliers for the subsequent calculation of the average and standard deviation values of each mechanical parameter for each group.

### 3.9. Mechanical Tests

The specimens obtained by injection molding were subjected to mechanical tests, including a minimum of 10 replicas of each test and compound. Tensile tests were performed according to ISO 527, using a LY-1065 testing machine (Dongguan Liyi Test equipment Co., Ltd., Dongguan, China). This machine was also used for the bending tests, according to ISO 178. In both cases, the loading application speed was fixed in 10 mm/min.

Regarding the impact tests, they were performed according to ISO 180/U (unnotched Izod method). The machine used was an IZOD&CHARPY Impact Tester model LY-XJJD 50 (Dongguan Liyi Test Equipment Co., Ltd., Dongguan, China), with a pendulum of 5.5 J and an impact speed of 3.5 m/s.

The results obtained for each mechanical parameter were statistically analyzed using the Kruskal–Wallis test with 1% significance level, as described in Section 3.8.

## 4. Conclusions

The present study reveals the feasibility of using vast fibers extracted from the invasive plant *P. setaceum* as reinforcement of polymeric material for injection molding. Based on the composition study of the natural fibers, 2% and 4% alkaline treatments enhanced the hydrophobic character of the fiber through increases of 32.9% and 42.7% of the cellulose content, respectively. In addition, with the application of the treatments, the initial thermal degradation temperature augmented in all cases, increasing up to 18.5% in the case of the combined alkaline and silane treatment.

Regarding the composites, the addition of untreated fibers did not significantly modify the values of tensile strength and modulus with respect to the unreinforced polymer (PE.Re). In the case of the PE.Re.PSt.20 composite, these values improved up to 16.8% and 40.5%, respectively, compared to the neat polymer. These increments are related to the 8.9% enhancement in crystallinity measured from the DSC test. In relation to the flexural behavior, the flexural modulus increased considerably (up to 74.7% with respect to the neat polymer) with the addition of 20 wt% of untreated fiber (PE.Re.PS.20). In this case, the composite with the best behavior was PE.Re.Pstd.40, which improved the tensile strength up to 69.2% and the flexural modulus up to 257.5%. The impact properties of the composites decreased up to 50.8% and 55.5% when 20 and 40 wt% of untreated fiber were added, respectively.

For all of the above, it is viable to convert the waste generated in the eradication campaigns of an invasive plant such as *P. setaceum* into the reinforcement of polymeric materials for injection molding, especially if chemical treatment based on alkaline and combined alkaline–silane treatments are used. This would allow for recovering part of the investment made to eliminate this species that is harmful to our environment, thus promoting the circular economy. In addition, it should be noted that this proposal of valorization is a high-added-value alternative, as this composite material with better tensile and flexural mechanical properties could be used in the production of multiple applications of injection molding. The addition of *P. setaceum* fibers not only replaces the corresponding amount of polymer in the composite, but also may imply a further material reduction if the enhanced tensile and flexural properties (achieved through correct chemical treatment) are exploited, thus minimizing the amount of plastic needed.

## Figures and Tables

**Table 1:** Composition of *Pennisetum setaceum* fibers before and after treatment.

Treatment	Individuals	Lignin (%)	Cellulose (%)	Hemicellulose (%)	Ash (%)
Untreated *P.* *setaceum* fibers (PS)	A	17.06 ± 0.34	37.37 ± 0.46	38.97 ± 1.22	9.89 ± 0.11
	B	14.82 ± 0.36	33.94 ± 1.18	38.47 ± 0.46	11.61 ± 0.14
	C	21.58 ± 2.13	35.90 ± 1.29	37.24 ± 0.48	12.62 ± 0.24
Acetic acid treatment (PStb)	A	18.08 ± 0.10	41.33 ± 0.39	42.13 ± 0.79	7.67 ± 0.20
Alkaline treatment (2%) (PSta)	B	14.23 ± 0.28	45.13 ± 0.09	30.16 ± 1.25	5.42 ± 0.09
Alkaline treatment (4%) (PSt)	C	14.45 ±0.12	51.23 ± 1.07	24.33 ± 0.93	5.66 ± 0.15
Silane treatment (PStc)	C	20.71 ± 0.63	36.38 ± 0.85	42.24 ± 1.71	12.91 ± 0.41
Alkaline + silane treatment (PStd)	C	14.47 ± 0.19	51.13 ± 0.09	26.66 ± 0.26	7.34 ± 0.16

**Table 2:** TGA results of untreated and treated *Pennisetum setaceum* fibers.

Fibers	T_i_ (°C)	T_max_ (°C)	Residue (%)
PS	194	348	28.62
PSt	219	363	22.75
PSta	212	366	22.33
PStb	211	364	20.29
PStc	212	374	22.87
PStd	230	373	22.98

**Table 3:** Differential Scanning Calorimetry results of composite and recycled high-density polyethylene.

Composite	Cooling		2nd Heating		Crystallinity (%)
	T_c_ (°C)	ΔH_c_ (J/g)	T_m_ (°C)	ΔH_m_ (J/g)	
PE.Re.PS.20	116.1 ± 0.9	−127.6 ± 13.3	134.6 ± 0.9	123.3 ± 15.0	52.6
PE.Re.PSt.20	115.0 ± 0.7	−135.6 ± 18.4	135.1 ± 0.7	139.0 ± 17.3	59.3
PE.Re.PSta.20	116.0 ± 0.9	−130.2 ± 5.7	134.8 ± 0.6	131.4 ± 6.2	56.1
PE.Re.PStb.20	116.2 ± 0.6	−130.5 ± 4.9	134.6 ± 0.5	127.6 ± 3.7	54.4
PE.Re.PStc.20	116.4 ± 0.9	−139.6 ± 21.6	134.4 ± 0.7	134.2 ± 20.4	57.3
PE.Re.PStd.20	115.5 ± 1.1	−136.3 ± 17.0	135.2 ± 1.0	133.3 ± 19.3	56.9
PE.Re.PS.40	114.8 ± 1.0	−96.9 ± 9.5 *	135.8 ± 1.0	97.5 ± 12.4 *	55.4
PE.Re.PSta.40	115.6 ± 0.8	−104.4 ± 10.0	135.4 ± 0.8	99.5 ± 9.6 *	56.6
PE.Re.PStb.40	116.8 ± 1.2	−103.7 ± 10.6	134.0 ± 0.9	101.3 ± 11.3	57.6
PE.Re.PStc.40	116.1 ± 0.8	−97.6 ± 5.4 *	134.8 ± 0.7	94.5 ± 7.3 *	53.8
PE.Re.PStd.40	115.8 ± 0.7	−92.3 ± 8.9 *	135.1 ± 0.7	87.9 ± 8.5 *	50.0
PE.Re	116.7 ± 0.2	−158.0 ± 10.5	134.3 ± 0.2	160.2 ± 9.7	54.7

* Statistically significant difference compared to PE.Re.

**Table 4:** Tensile test results of the composites and unreinforced recycled high-density polyethylene.

Composite	Tensile Strength (MPa)	Elongation at Maximum Tensile Strength	Tensile Modulus (MPa)
PE.Re.PS.20	20.51 ± 0.24	0.0824 ± 0.0027	782.06 ± 64.04
PE.Re.PSt.20	24.15 ± 0.33 *	0.0738 ± 0.0026 *	979.41 ± 37.43 *
PE.Re.PSta.20	23.92 ± 0.28 *	0.0755 ± 0.0022	989.91 ± 9.45 *
PE.Re.PStb.20	23.10 ± 0.13	0.0768 ± 0.0027	936.69 ± 31.48 *
PE.Re.PStc.20	23.59 ± 0.19 *	0.0748 ± 0.0037	1015.84 ± 88.56 *
PE.Re.PStd.20	23.76 ± 0.17 *	0.0781 ± 0.0027	912.99 ± 61.57 *
PE.Re.PS.40	20.45 ± 0.12	0.0415 ± 0.0022 *	800.64 ± 131.83
PE.Re.PSta.40	21.89 ± 0.33	0.0418 ± 0.0015 *	1001.18 ± 80.79 *
PE.Re.PStb.40	20.44 ± 0.08	0.0385 ± 0.0015 *	878.90 ± 93.86
PE.Re.PStc.40	21.52 ± 0.16	0.0394 ± 0.0018 *	951.61 ± 88.11 *
PE.Re.PStd.40	22.02 ± 0.15	0.0407 ± 0.0014 *	952.47 ± 45.15 *
PE.Re	20.66 ± 0.20	0.1345 ± 0.0092	697.04 ± 39.65

* Statistically significant difference compared to PE.Re.

**Table 5:** Flexural test results of the composites and unreinforced recycled high-density polyethylene.

Composite	Flexural Strength (MPa)	Elongation at Maximum Flexural Strength	Flexural Modulus (MPa)
PE.Re.PS.20	32.48 ± 0.61	0.0654 ± 0.0017	1550.01 ± 47.53
PE.Re.PSt.20	40.17 ± 0.08 *	0.0641 ± 0.0013 *	2042.33 ± 44.41 *
PE.Re.PSta.20	37.15 ± 0.40	0.0651 ± 0.0012	1927.27 ± 66.07
PE.Re.PStb.20	38.12 ± 0.30	0.0664 ± 0.0018	1956.01 ± 49.35
PE.Re.PStc.20	38.22 ± 0.49 *	0.0661 ± 0.0011	1878.14 ± 42.74
PE.Re.PStd.20	38.26 ± 0.57 *	0.0657 ± 0.0019	1920.80 ± 63.95
PE.Re.PS.40	38.92 ± 0.89 *	0.0456 ± 0.0034 *	2875.32 ± 46.21 *
PE.Re.PSta.40	42.93 ± 0.71 *	0.0409 ± 0.0036 *	3034.69 ± 19.01 *
PE.Re.PStb.40	41.28 ± 0.54 *	0.0428 ± 0.0029 *	2968.41 ± 118.16 *
PE.Re.PStc.40	42.76 ± 0.36 *	0.0419 ± 0.0026 *	2959.78 ± 86.16 *
PE.Re.PStd.40	43.78 ± 0.68 *	0.0432 ± 0.0036 *	3170.75 ± 72.22 *
PE.Re	25.88 ± 0.29	0.0772 ± 0.0011	886.85 ± 33.84

* Statistically significant difference compared to PE.Re.

**Table 6:** Impact test results of the composites and unreinforced recycled high-density polyethylene.

Composite	Impact Strength (kJ/m^2^)
PE.Re.PS.20	14.91 ± 0.92
PE.Re.PSt.20	13.52 ± 1.12
PE.Re.PSta.20	13.34 ± 0.81
PE.Re.PStb.20	12.59 ± 1.21 *
PE.Re.PStc.20	13.33 ± 1.04
PE.Re.PStd.20	13.48 ± 1.08
PE.Re.PS.40	13.49 ± 0.10
PE.Re.PSta.40	8.58 ± 0.75 *
PE.Re.PStb.40	6.97 ± 0.39 *
PE.Re.PStc.40	7.36 ± 0.51 *
PE.Re.PStd.40	8.13 ± 0.82 *
PE.Re	30.32 ± 0.34

* Statistically significant difference compared to PE.Re.

**Table 7:** Abbreviations of treatments.

Treatment	Abbreviation
Alkaline treatment 4% (1 h)	t
Alkaline treatment 2% (1 h)	ta
Acetic acid treatment 10% (2 h)	tb
Silane treatment 3% (1 h)	tc
Alkaline treatment 4% (1 h) + silane treatment 3% (1 h)	td

## Data Availability

All data generated or analyzed during this study are included in this published article.

## Supplementary Materials

The following supporting information can be downloaded at: https://www.mdpi.com/article/10.3390/plants12091777/s1, Figure S1: Tensile, flexural, and impact strength of the composites and unreinforced recycled high-density polyethylene. Figure S2: Modulus of elasticity (tensile and flexural) of the composites and unreinforced recycled high-density polyethylene.
