# Supplementary material for: Valorization of Pennisetum setaceum: From Invasive Plant to Fiber Reinforcement of Injected Composites
Source: Plants (Basel). 2023 Apr 26;12(9):1777. doi: 10.3390/plants12091777 (PMC10181305; doi:10.3390/plants12091777)
Supplement: Supplementary file 1 [file plants-12-01777-s001.zip › plants-2279276-supplementary.pdf]

plants

# Supplementary Materials

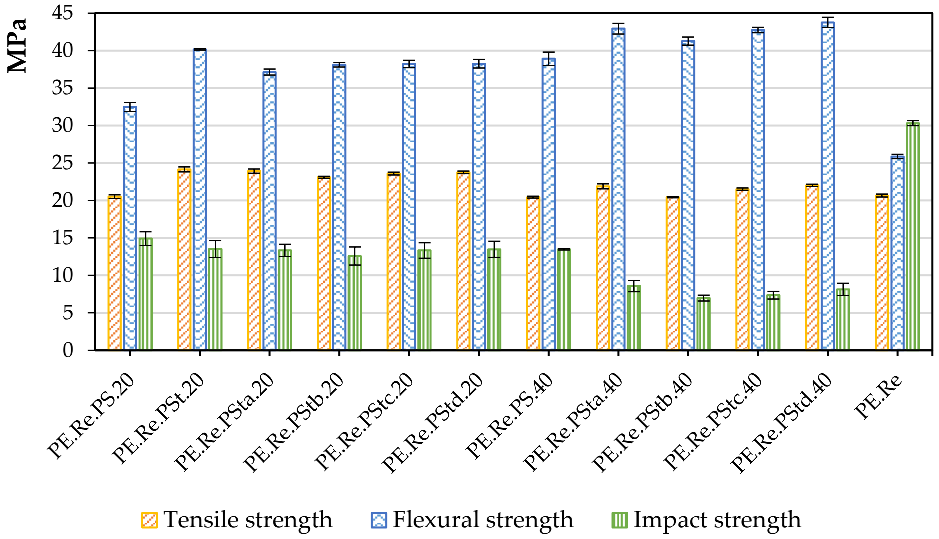


**Figure S1.** Tensile, flexural and impact strength of the composites and unreinforced recycled high-density polyethylene.

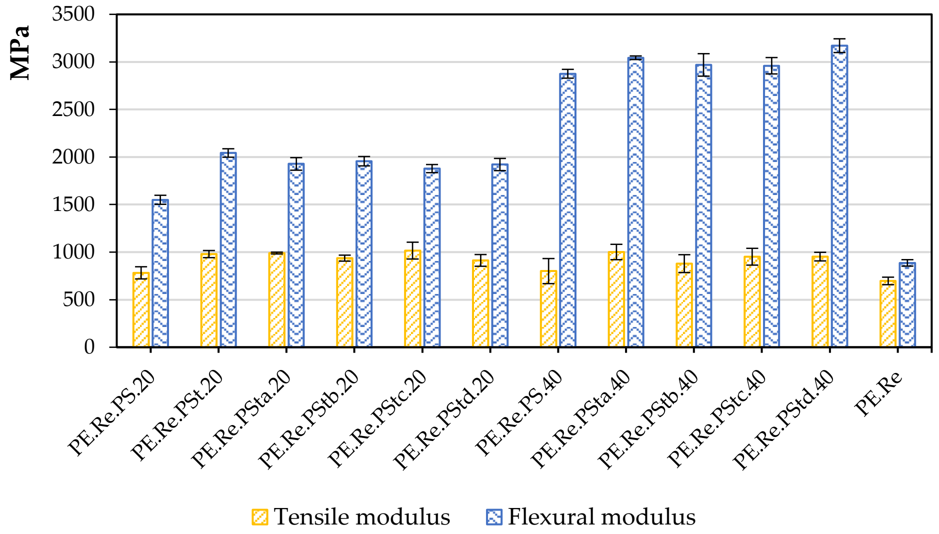


**Figure S2.** Modulus of elasticity (tensile and flexural) of the composites and unreinforced recycled high-density polyethylene.
